# Supplementary material for: Live observation of the oviposition process in Daphnia magna
Source: PLoS One. 2019 Nov 4;14(11):e0224388. doi: 10.1371/journal.pone.0224388 (PMC6827901; doi:10.1371/journal.pone.0224388)
Supplement: Attachment — Submitted filename: response (2, Plos One).docx [file pone.0224388.s005.docx]

Reviewer #1: While the scope is narrow, this descriptive morphological study revealed some interesting details concerning the process of egg deposition in daphnia. The following are detailed comments.

1 Some statements regarding basic daphnia biology are not accurate. Second paragraph of introduction, during parthenogenic reproduction, *Daphnia magna* can produce up to ~80 neonates depending on availability of food, ambient temperatures, age, etc. The range of 20-30 is too narrow. The authors are suggested to check with the classic review by Hebert (1978). Third paragraph of introduction, the brood interval is not fixed at 3 days but varies depending on several factors.

We are grateful of helpful comments of the reviewer. We carefully took a look at the review by Hebert (1978) and corrected as suggested. Ebert (2005) also indicated that an adult female may produce a clutch of eggs every 3 to 4 days until her death.

2. Third paragraph of results, each ephippium is known to contain two resting eggs. Why one resting egg?

We understand that each ovary produce one resting egg. Since an ephippium originate from two ovaries, it contains two resting eggs. The text was corrected as suggested.

3. The first sentence of the 4th paragraph needs to be backed up by references. So, according to this statement egg activation occurs before vitelline envelope formation? Also, what did the authors exactly mean by egg activation?

As suggested, we backed up the sentence with two references (Anderson, 1967; Masuda et al., 1991). The most critical event of egg activation may be the elevation of intracellular calcium levels (Horner and Wolfner, Dev Dyn 237:527-544, 2008). Egg activation in *Drosophila* is known to occur during ovulation (Kaneuchi et al., PNAS 112:791-796, 2015). Egg activation of shrimp was also reported during spawning and formation of vitelline envelope follows after egg activation (Pongtippatee-Taweepreda et al, 2004; Pongtippatee et al., 2012). VE formation is followed after egg activation (Anderson, 1967; Masuda et al., 1991).

4. Second last sentence of the 4th paragraph in the results section, “to form after approximately 20 min” is not clear. It meant to be “approximately 20 min after egg deposition”?

I appreciate for your correction. We changed the sentence as suggested.

5. Second sentence of the third paragraph in discussion, saying “arthropod eggs” is too general. Consider “the eggs of some arthropods”. Same paragraph, “The VE formed within…….”.

I appreciate for your careful reading. We changed the sentence as suggested.

6. There does not appear to be good logic in the last 4 sentences of the 3rd paragraph in discussion. There is a lack of causal relationship between the three sentences concerning shrimp and prawn and the last sentence. What does a delay in VE formation observed in a shrimp and a prawn have anything to do with the speculation that egg activation in daphnia is triggered by squeezing in the oviduct?

It is likely that egg activation in *Daphnia* also precedes VE formation. We speculate that Daphnid eggs are activated during oviposition which is the most visible event prior to VE formation. In fact, *Drosophila* eggs are activated by pressure when they pass through the reproductive tract (Horner and Wolfner, 2008). We carefully rewrote the sentences to emphasize our points.

7. References are not standardized. Each item should be in the same format and the Latin names italicized.

We formatted the whole manuscript, following strictly the instruction of Plos One. We also standardized the list of references as suggested.

Reviewer #2: This article studied the oviposition process and egg activation of Daphnia magna from the perspective of morphology. This study is not suitable for submission to this journal and is recommended for submission to professional morphology journals.

We admit that this manuscript is descriptive. However, our works provide an insight how a parthenogenic egg starts development after oviposition. It is known that developmental patterns of embryos in many species are already fixed in oocytes. For example, the anterior-posterior and dorsal-ventral axes of *Drosophila* embryos are determined during oogenesis. Even if *Drosophila* eggs pass through a tight reproductive track for ovulation, their cytoplasmic arrangement should not be disturbed. However, the oviposition pattern of the parthenogenic *Daphnia* egg does not allow us to imagine that the developmental axis are maintained during oviposition. What we observed is that the egg contents flow through a narrow egg canal. After oviposition, *Daphnia* eggs are known to follow a strict developmental pattern. Significance of this work may be that we raise a possibility that developmental axis of *Daphnia* may not be fixed during oogenesis but be established after oviposition. Since our observations provide an insight on a new way for developmental pattern formation, we believe that this manuscript brings a general interest in the field of animal development as well as *Daphnia* research.
